# Morphology and niche evolution influence hummingbird speciation rates

**DOI:** 10.1098/rspb.2022.1793

**Published:** 2023-04-26

**Authors:** Elisa Barreto, Marisa C. W. Lim, Danny Rojas, Liliana M. Dávalos, Rafael O. Wüest, Antonin Machac, Catherine H. Graham

**Affiliations:** ^1^ Swiss Federal Institute for Forest, Snow and Landscape Research WSL, Zürcherstrasse 111, 8903 Birmensdorf, Switzerland; ^2^ Departamento de Ecologia, Universidade Federal de Goiás, Campus Samambaia, Goiânia, Goiás, Brazil; ^3^ Department of Ecology and Evolution, Stony Brook University, 650 Life Sciences Building, Stony Brook, NY 11794, USA; ^4^ Department of Natural Sciences and Mathematics, Pontificia Universidad Javeriana Cali, Cl. 18 #118-250, Cali, Valle del Cauca, Colombia; ^5^ Consortium for Inter-Disciplinary Environmental Research, Stony Brook University, 129 Dana Hall, Stony Brook, NY 11794, USA; ^6^ Villum Center for Global Mountain Biodiversity and Center for Macroecology, Evolution and Climate, GLOBE Institute, University of Copenhagen, Universitetsparken 15, 2100 Copenhagen, Denmark; ^7^ Center for Theoretical Study, Charles University and the Czech Academy of Science, Jilska 1, 11000 Prague, Czechia; ^8^ Department of Ecology, Charles University, Vinicna 7, 12844 Prague, Czechia

**Keywords:** dynamic traits, diversification, evolutionary divergence, niche conservatism, Trochilidae

## Abstract

How traits affect speciation is a long-standing question in evolution. We investigate whether speciation rates are affected by the traits themselves or by the rates of their evolution, in hummingbirds, a clade with great variation in speciation rates, morphology and ecological niches. Further, we test two opposing hypotheses, postulating that speciation rates are promoted by trait conservatism or, alternatively, by trait divergence. To address these questions, we analyse morphological (body mass and bill length) and niche traits (temperature and precipitation position and breadth, and mid-elevation), using a variety of methods to estimate speciation rates and correlate them with traits and their evolutionary rates. When it comes to the traits, we find faster speciation in smaller hummingbirds with shorter bills, living at higher elevations and experiencing greater temperature ranges. As for the trait evolutionary rates, we find that speciation increases with rates of divergence in the niche traits, but not in the morphological traits. Together, these results reveal the interplay of mechanisms through which different traits and their evolutionary rates (conservatism or divergence) influence the origination of hummingbird diversity.

## 1. Introduction

Diversification rates, which encompass both speciation and extinction rates, vary dramatically over time, and across regions and taxa. However, the interplay of factors that contribute to this variation remains largely unresolved [1,2]. Speciation has been associated with species' morphological and niche traits [3,4], but also with the rates at which these traits evolve over time [5,6]. Currently, it is unclear whether it is the traits and/or the rates at which they evolve (also referred to as static and dynamic traits [2]) which influence speciation. Further, there are two possibilities as to how the rate of trait evolution can influence speciation. Speciation can be promoted by trait divergence (fast evolutionary rates) and/or by trait conservatism (slow evolutionary rates) [1,7]. To test these ideas, we evaluate a series of related hypotheses using multiple morphological and niche traits in hummingbirds, a clade of non-passerine birds known for diversifying across a range of climates (from the tropical to the temperate) and elevations (Amazonian lowlands to the peaks of the Andes), and with a variety of bill and body sizes.

Morphological traits, especially those that are key to an individuals’ survival and reproduction, such as those associated with resource acquisition, competition, metabolic rates and dispersal, can influence speciation rates and, by extension, diversification rates [2]. Two such traits in hummingbirds are body mass and bill length. Body mass is related to hummingbird size, abundance, metabolic rates and thermoregulatory requirements [8–10]. Small-bodied species may diversify faster because they tend to have larger populations, lower energetic needs and shorter generation times, all of which can increase the chance of speciation while minimizing extinction risk [11,12]. Bill length influences competition, access and efficiency when feeding on floral nectar resources [13–15]. Hummingbirds with shorter bills tend to be more generalists in their interactions with plants [16,17], which makes them more resilient to variation in resource availability and is also likely to facilitate range expansion and exploration of novel habitats and resources. As a result, we expect negative associations between hummingbird speciation rates and their body mass and bill length.

In addition to the morphological traits, speciation rates can be influenced by niche dimensions, such as those that describe the climatic and topographic preferences of a species [4,18,19]. These abiotic niche traits are hard to measure directly but can be approximated from species distributions that roughly capture the range (i.e. niche breadth) and average value (i.e. niche position) of the environmental conditions where a species occurs [4,19]. Niche breadth has been found to relate both negatively and positively to diversification rates, depending on the underlying mechanism that is presumably at play [19,20]. Species with wider niches tend to have larger geographical ranges, which increases the chances of allopatric speciation [21,22]. They also tend to be more resilient to environmental change, which reduces extinction [19,21,22]. The opposite relationship is also plausible, as species with wide niches might diversify slowly because they are spread over a larger geographical area and are subject to greater gene flow and therefore decreased speciation [4,19,21]. The effects of niche position on diversification may depend on the environmental factor under investigation. Topographic affinity, for example, could have mixed effects on diversification. Lowland species might diversify faster because they live in highly productive regions with high temperatures [23], but they also compete with the high richness of other lowland species, which could suppress or spur their speciation [18]. Highland species, on the other hand, occur in smaller geographical areas with lower temperatures and oxygen availability, which might slow down their diversification [18,23]. Current evidence for hummingbirds is mixed highlighting the need for further investigation [18,24].

It is also possible that speciation rates are more strongly affected by the rates of trait evolution than by the traits themselves [2,25]. Two contrasting processes may mediate the link between speciation rates and the rates of trait evolution: evolutionary divergence or conservatism [26]. Evolutionary divergence may foster diversification because when species diverge faster along the axes of their trait or niche space, they may discover and exploit new resources and adapt to novel conditions [27,28]. Extinction would then decrease, and speciation would increase, as individuals disperse and diverge from the parent population [6,29,30], increasing the possibility of adaptive radiation [28]. For example, diversification has been associated with clade divergence along an elevational gradient [31]. Conversely, the conservatism hypothesis postulates that, as species retain their ancestral characteristics through time and fail to adapt to novel conditions (e.g. changes in climate or resource availability), their populations become fragmented, and gene flow decreases, resulting in speciation [7]. Speciation through population fragmentation across comparable environments has been reported to produce non-adaptive radiations, at least in some taxa [32].

Trait- and rate-based hypotheses are not mutually exclusive, and both have been supported by the literature [6,19] but rarely tested in conjunction [25]. Here, we test the effects of (i) multiple classes of traits (morphology, climate characteristics and elevation), (ii) the evolutionary rates of these traits and (iii) trait conservatism and divergence on speciation rates. Hummingbirds show considerable variation in their morphology, climatic and elevational preferences, but also strong physiological constraints imposed by their fast metabolism, hovering flight and nectarivory [33]. Hummingbirds vary markedly in their diversification rates [34], which makes them an excellent system to parse out the effects of traits, evolutionary divergence and conservatism. We find that the tested effects are not necessarily exclusive; some types of traits directly influence speciation, while others act through their evolutionary rates [2]. Similarly, divergence might promote speciation in some traits, but conservatism in others. Together, these findings elucidate the rich interplay of pathways through which traits might influence speciation in hummingbirds.

## 2. Material and methods

### (a) Overall approach

We compiled species-level data for hummingbirds on their body mass, bill length, mid-elevation, and the breadth and position of their temperature and precipitation preferences. Moreover, we calculated present-day rates of evolution for each of these characteristics using the Bayesian analysis of macroevolutionary mixtures (BAMM) [35] and estimated present-day speciation rates. We focus primarily on the speciation rates near the tips because these rates can be most reliably estimated from present-day phylogenies (e.g. [36]). We used multiple methods to estimate speciation rates and relate them to traits and their evolutionary rates, because each method has its strengths and weaknesses, as we detail later. Similar results obtained from multiple methods were interpreted as indicative of robustness and considered strong evidence of an effect, while results obtained only once or very few times were considered weaker evidence. A flow chart summarizing the analyses are available in electronic supplementary material, figure S1.

### (b) Traits

**Figure 1.**
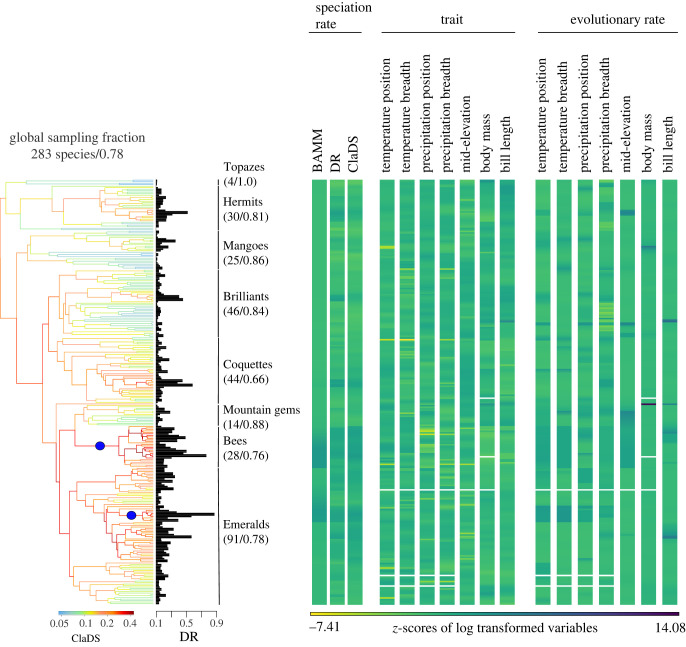
Hummingbird phylogeny pruned from McGuire *et al*. [34] comprising 283 species and the associated data on speciation rates estimated by BAMM, DR and ClaDS, morphological and niche traits (climate and elevation) and the evolutionary rates of these traits estimated by BAMM (data available in electronic supplementary material, appendix S1). All variables on the right panel were log-transformed and standardized to mean 0 and standard deviation of 1 (*z*-score). Blanks indicate missing data. Hummingbird clades are indicated in the phylogeny with their corresponding sampling fraction in parentheses (absolute number and proportion of species included in the study). The phylogeny is coloured by speciation rates estimated by ClaDS, with the rate shifts estimated by BAMM indicated by blue dots. The bars in front of each tip of the phylogeny illustrate speciation rates calculated by the DR index. A similar figure for an alternative phylogenetic hypothesis [38] is in the electronic supplementary material, figure S5. The heatmap and the barplot were created using the phytools R package [39].

We chose traits that were available across most hummingbird species and had well supported hypotheses in the literature as to how they could influence speciation rates. For morphology, we chose body mass and bill length, because these two traits relate to multiple aspects of hummingbird's life history, physiology, and mutualistic and antagonistic interactions [10,15,16]. Bill curvature is also important to determine species interactions among hummingbirds and consequentially, their competition, niche partitioning and coexistence, all of which could influence speciation rates [16]. However, this information is lacking for many species. We used data on hummingbird mean body mass collected by D. Rojas and mean exposed bill length from a published dataset [37] (figure 1; electronic supplementary material, appendix S1). Our measurements of body mass were highly correlated with those from [37] (Pearson's *r* = 0.96, electronic supplementary material, figure S2) but included six to eight additional species depending on the phylogeny [34,38]. Intraspecific variation in morphological traits is unlikely to bias the results, given that the coefficient of variation (CV) of both traits was four to five times greater between than within species (bill length intraspecific CV = 6.44% and interspecific CV = 34.28%; whereas body mass intraspecific CV = 12.81% and interspecific CV = 52.11%).

For niche traits, we chose two characteristics of the realized climatic niche that are hypothesized to relate to diversification: breadth and position (figure 1; electronic supplementary material, appendix S1). Niche breadth describes how much of the climate niche space is occupied, while niche position quantifies which part of the niche is typically occupied. For niche breadth, we calculated the difference between the maximum and minimum values (i.e. range). For niche position, we calculated the median value of temperature and precipitation within the range of each species (mean value was strongly correlated with the median; electronic supplementary material, figure S3). We chose two climatic variables, one related to temperature and another to precipitation. We calculated mean diurnal temperature range (Bio2; hereafter referred to as temperature) and annual mean precipitation (Bio12; hereafter referred to as precipitation) at 30 arc sec resolution from the CHELSA database [40] within each species geographical distribution using the ‘raster’ package in R [41]. We chose diurnal temperature range to capture the daily fluctuation that is experienced by species and to avoid using temperature variables that are highly correlated with elevation. We used data on the geographical distribution of species from expert range maps [42] which is available for more hummingbird species than alternative distributional data [43] and results in more conservative estimates of niche breadth (electronic supplementary material, figure S4).

Speciation rates is also likely to vary in response to biogeographical factors, such as topography [23,24]. Thus, we calculated elevational mid-points by taking the mean of maximum and minimum elevation for each species, which was derived by D. Rojas mostly from expert range maps [42] (figure 1; electronic supplementary material, appendix S1). These data are strongly correlated with those published for all birds [23] (Pearson's *r* = 0.95, electronic supplementary material, figure S2), but include more hummingbird species (8 to 10 additional species depending on the phylogeny).

### (c) Phylogeny, diversification rates and the rates of trait evolution

We estimated speciation rate and the rate of trait evolution at the tips of the time-calibrated phylogenetic tree for each morphological and niche trait using the two most complete maximum clade credibility hummingbird phylogenies available [34,38]. The final dataset contained data for 283 species when using [34] and 233 using [38] phylogeny, the latter excluding species with no genetic information.

We estimated species-level speciation rates using two model-based (i.e. BAMM [35] and cladogenic diversification rate shift—ClaDS [44,45]) and one model-free (i.e. diversification rate—DR [38]) approach, as there is no consensus on the best method to estimate such rates [46,47]. The DR index is based on phylogenetic branching patterns and is a rough estimate of speciation rates under a pure-birth model [36,38], but has been shown to underperform when compared to BAMM [48]. The ClaDS method focuses on estimating both small and large shifts in diversification rates and is likely to detect more heterogeneous rates among lineages than BAMM [44]. We estimated speciation rates using ClaDS2 model with data augmentation with ‘jPANDA’ package in Julia [44,45].

We further estimated rates of speciation and trait evolution using BAMM v.2.5.0 [35] with the ‘BAMMtools’ R package to generate the control file (including priors) and to extract the results [49]. We chose BAMM to estimate trait evolution because it outperforms other methods [48]. For diversification analysis in BAMM, we set the rate shift prior to *γ* = 1 and sampled the models every 1000 generations for a total of 11 million generations using a burn-in of 1 million generations. The number of generations and burn-in used to estimate traits evolutionary rates varied and were chosen based on the visual inspection of the log-likelihood trace of the MCMC output and the effect sample sizes (greater than or equal to 200). We accounted for incomplete species sampling when estimating speciation rates using ClaDS and BAMM by informing the clade sampling fraction of each of the phylogenetic trees according to the taxonomy adopted by the IOC World Bird List v.12.2 [50] (figure 1; electronic supplementary material, figure S5). The DR index does not accommodate information on missing species, which could lead to biased estimates of speciation.

### (d) Evaluating the effects of traits and trait rates on speciation

Given the lack of consensus in the literature as to which is the most accurate method to test for correlates of speciation rates (e.g. [48,51,52]), we tested univariate relationships with three semiparametric tip-rate correlation methods and multivariate relationships with two multi-predictor regressions that account for phylogenetic non-independence. Further, given the lack of intraspecific data across all traits (i.e. climatic niche and elevation), we did not explicitly account for this source of variation in the statistical analysis.

Univariate correlations were tested using the (i) correlated speciation and trait rates simulation (Cor-STRATES), which has a good power to detect associations between rates and is robust to measurement errors [48]. Cor-STRATES consists of calculating Spearman's correlation coefficient between speciation rates and trait rates estimated from a tree rescaled by Pagel's lambda [53]. Next, this empirical correlation coefficient is compared to a null distribution of coefficients using a two-tailed test. The null distribution comes from correlating speciation with trait rates obtained from simulated traits evolving under Brownian Motion on the original tree. All steps were performed with phytools and geiger R packages [39,54]. We implemented Cor-STRATES using only speciation rates from BAMM and ClaDS because simulations with Cor-STRATES show the DR index does not perform well [48]. In addition, we used the same simulated traits to test for a correlation between speciation rates and trait values. (ii) We used the DR index in a slightly modified version of the inverse equal splits simulation tests (ES-sim) by calculating the inverse of the mean equal splits measure (ES), so it becomes DR [52]. ES-sim simulates neutral trait evolution and correlates it with the DR index multiple times (we ran 1000 replicates) to generate a null distribution to which the empirical correlation is compared [52]. (3) We used the structured rate permutations on phylogenies (STRAPP) method to test for univariate associations between speciation and trait values or trait evolutionary rates using estimates from BAMM [51]. This semiparametric method was specifically designed for BAMM estimates and compares the empirical association to a null distribution built from permuting trait values across the different diversification regimes identified by BAMM [51]. We did not consider these results in the main text because of STRAPP's low power for phylogenies with fewer than 800 tips and with little variation in diversification regimes [51], which is the case for hummingbirds (figure 1; electronic supplementary material, figure S5) [55]. All results from STRAPP can be found in electronic supplementary material, figure S6 and table S1.

These simulation methods are robust ways to test correlates of speciation rates (e.g. [47,55]), but unfortunately they test only univariate relationships, whereas speciation rates are more likely influenced by multiple factors [2]. Therefore, we also fitted two classes of multi-predictor regressions in which ClaDS speciation rate is the response variable (*y*) and all seven traits and their respective evolutionary rates are the predictor variables (*x*). We did not use speciation rates estimated by BAMM as response variables in these 14-predictor regressions because the lack of statistical independence on rates estimated within a regime can bias the results [56]. Specifically, we fitted (iv) Phylogenetic generalized least-squares (PGLS) regressions using caper R package [57] with the lambda transformation parameter estimated by maximum likelihood and (v) phylogenetic Bayesian generalized linear mixed model (GLMM) with the MCMCglmm R package [58]. The phylogenetic GLMM account for phylogenetic relatedness by fitting species identity as correlated random factors whose covariation depends on the phylogeny topology. Species were set as the random effects. We ran each model with 1 million iterations, a burn-in of 5000, and a thinning rate of 1000 and ensured that the models had ESS > 200 and all levels converged ($\hat{R}<1.1$). For random effects and residual variance, we used an inverse-Wishart prior (*V* = 1, *nu* = 0.02). All predictors were standardized using *z*-scores (units of standard deviation) prior to the analyses for comparison of their relative importance. We accounted for multicollinearity by ensuring that no predictor had a Pearson's correlation coefficient higher than |0.7| [59] (electronic supplementary material, figure S7). Removing the heavy Giant hummingbird (*Patagona gigas*) and the long-billed Sword-billed hummingbird (*Ensifera ensifera*) from models of body mass and bill length did not change the results (electronic supplementary material, tables S1–S4). The R script used in this study is available in the electronic supplementary material.

## 3. Results

**Figure 2.**
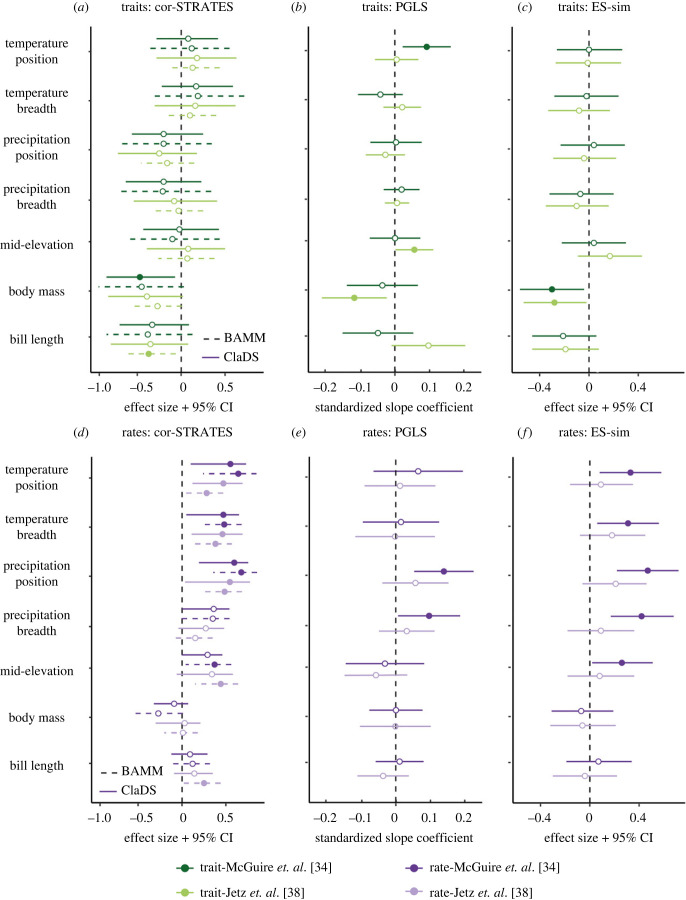
Estimated relationships between (*a–c*) traits and (*d–f*) their evolutionary rates with speciation rates of hummingbirds. Dots are effect sizes and lines are either 95% confidence intervals (*a,c,d,f*) or standard errors (for PGLS results, *b* and *e*). Filled dots indicate 95% confidence intervals that does not include zero. Relationships between speciation rates and trait values are indicated by shades of green (first row) and trait evolutionary rates by shades of purple (second row). These results were obtained using two alternative phylogenies (darker colours refer to results using the phylogeny [34] and lighter colours to [38]), three methods to estimate speciation rates (BAMM, ClaDS and DR), and three statistical approaches to correlate speciation rates to traits and/or rates (*a,d*: Cor-STRATES, *b,e:* PGLS, and *c* and *f*: ES-sim)—see the methods section for more details. Effect sizes from PGLS are often smaller than from other tests because it corresponds to standardized slope coefficients, whereas all other effect sizes correspond to correlation coefficients. The complete set of results is available in electronic supplementary material, tables S2–S4.

Because of the controversy about which is the most accurate estimate of speciation rate and how to better correlate such rates to traits and evolutionary rates (e.g. [48,51,52]), we decided to look for consistency across alternative approaches. We found that the correlation between speciation rates estimated by the three different methods and the two alternative hummingbird phylogenies are intermediate to high (BAMM and ClaDS: Pearson's *r* = 0.79 and 0.66; BAMM and DR: 0.57 and 0.55; ClaDS and DR: 0.70 and 0.64, respectively, for the phylogenies [34] and [38]; figure 1; electronic supplementary material, figure S5 and S8). Yet, we found considerable variation in the effect size of the relationships depending on the method used to estimate speciation rates, the statistical approach to relate it to the predictors, and the phylogeny (figure 2; electronic supplementary material, tables S1–S5). The two multi-predictor regression models (PGLS and GLMM) yielded similar results, which is expected given their analytical similarities. Therefore, we only present and discuss in the main text the coefficients estimated from the PGLS (see electronic supplementary material, table S5 and figure S9 for the GLMM results). The PGLS 14-predictor models had a low explanatory power (*R*²), ranging from 0.07 to 0.09 (electronic supplementary material, table S4). Despite variation among the alternative approaches, we found a general pattern of speciation rates being more frequently associated with rates of niche evolution than with the niche itself, with a trend towards positive associations between both rates (purple coefficients in figure 2; electronic supplementary material, tables S1–S5). The opposite is true for morphology, whose traits themselves, rather than their evolutionary rates, were more often correlated with speciation rates and mostly negatively (green coefficients in figure 2; electronic supplementary material, tables S1–S5). Below we summarize results for traits and their evolutionary rates across all alternative approaches.

### (a) Speciation as function of traits

We found evidence that body mass and bill length are negatively associated with speciation rates, meaning that smaller and shorter billed hummingbirds speciate more quickly (figure 2*a–c*; electronic supplementary material, tables S2–S5). Although such negative association was identified for most tests, only a subset had confidence intervals not intercepting zero (filled dots in figure 2*a–c*). Those were four correlation coefficients for body mass that ranged from −0.47 to −0.27 (one from Cor-STRATES, two from ES-sim and one from the PGLS) and one coefficient of −0.34 for bill length from Cor-STRATES (figure 2*a–c*; electronic supplementary material, tables S2–S5). Faster speciation rates were also found among hummingbirds that inhabit higher elevations and experience greater daily temperature ranges, but only when fitting multi-predictor regressions (filled dots in figure 2*b*; electronic supplementary material, tables S4–S5). No other trait tested had a clear association with hummingbird speciation rates (figure 2*a–c*).

### (b) Speciation as function of trait evolutionary rates

We found a trend of positive associations between tip-rate speciation and evolutionary rates for all niche traits and, for a few tests, for bill length evolution (coefficients skewed to the right side of the vertical dotted line in figure 2*d–f*; electronic supplementary material, tables S2–S5). This positive trend suggests that faster changes in traits over the hummingbird's evolutionary history are associated with increased speciation, as expected from the evolutionary divergence hypothesis. Positive associations whose confidence interval does not include zero were observed across different estimates of speciation rates (i.e. DR, BAMM and ClaDS), statistical tests (i.e. Cor-STRATES, ES-sim, PGLS/GLMM) and phylogenies (filled dots in figure 2*d–f*; electronic supplementary material, tables S2–S5), except for the ES-sim and the PGLS/GLMM tests when using data derived from the phylogeny with fewer species (figure 2*e,f*; electronic supplementary material, tables S2–S3).

## 4. Discussion

We evaluated a series of hypotheses involving trait and trait divergence and conservatism in hummingbirds by using different but equally valid statistical approaches to estimate speciation rates and its correlates. Given the lack of consensus on a single best approach to address these questions, we considered the frequency with which a result emerged from the different tests to be a measure of strength of evidence. We found that hummingbird speciation is associated with morphological and niche traits, whereby speciation tends to be faster in smaller and, based on weaker evidence (i.e. this results only appears in few tests), in short-billed species that live at high elevations and face greater variation in daily temperature. By contrast, we found no clear effect of niche breadth (in precipitation and temperature) on speciation, even though hummingbirds vary markedly in their geographical ranges, spanning a variety of precipitation and temperature regimes (e.g. from the cloud forests in the Andes to the dry open areas in the Cerrado [43]). In addition, our results revealed a consistent trend of evolutionary divergence in niche promoting speciation in hummingbirds [29,48,60]. Hummingbirds whose environmental niches (temperature, precipitation and elevation) and bill length change faster had greater speciation rates, although the effect of bill length evolution on speciation rates was only detected by one test. While the results varied to some extent across methods and hummingbird phylogenies [34,38], the main conclusions were largely robust (figure 2). In general, hummingbird speciation rates are related to their morphology and the speed at which their niches evolve, but not necessarily with morphological evolution or the niches themselves. Together, our findings begin to unravel the interplay of morphological traits, niche traits, their rates of evolution, and conservatism and divergence on hummingbird speciation.

### (a) Speciation correlates with hummingbird morphology

Several morphological traits are reportedly correlated with diversification in birds, including body size, wing morphology and brain size [61–63]. Among hummingbirds, we found evidence that smaller and shorter billed species have the highest rates of speciation (figure 2*a–c*). The potential role of body mass on speciation rates appears contradictory with the lack of association between body size on species richness found across multiple avian families [3,61]. However, correlates of rates of speciation and diversity itself may not always be the same, emphasizing the importance of evaluating both rates and richness. Body size also seems unrelated to diversification of other large groups, such as mammals and squamates [11,12], suggesting that the link between body size and diversity could be group-dependent and vary with taxonomic scale. In the case of hummingbirds, body mass and bill length are tightly related to biotic interactions and foraging behaviour. Many smaller-bodied hummingbirds are generalists [64] and have opportunistic foraging strategies that do not require defending a floral resource (low-reward trapliners, filchers), which could facilitate coexistence [13]. Smaller species with shorter bills can also more easily take advantage of primarily insect-pollinated flowers that produce lower nectar yields and tend to have wider corollas, which are not frequently visited by larger hummingbirds [13,16]. This foraging behaviour could favour shifts from bee to hummingbird pollination, especially at high elevations, where flower visitation by insects is less frequent owing to their physiological constraints [65]. Thus, hummingbirds that can feed on such flowers may experience reduced competition in a new adaptive zone, all of which can promote diversification and trait evolution [66].

### (b) Varying associations between speciation and niche traits

Hummingbird richness varies considerably across environments, with richness generally being lower at very high elevations or cool, dry environments than in low to mid-elevations or warm, wet environments [43]. These elevational and climatic conditions also influence hummingbird community structure [67], and are thus expected to relate to diversification. However, we found only week evidence that hummingbirds that typically experience higher daily ranges of temperatures and live at higher elevations diversify faster, since these relationships only emerged from the multivariate tests (figure 2*b*). Meanwhile, precipitation preferences and temperature niche breadth had no clear effect on speciation rates (figure 2*a–c*). The positive association between daily temperature range and speciation found in hummingbirds supports the hypothesis that species that tolerate broader changes in environmental conditions are more resilient to environmental change [19] and/or more likely to have broader ranges and experience allopatric speciation [21,22]. However, we did not find a clear pattern when it comes to precipitation, perhaps because it has a less direct influence on hummingbird physiology and instead acts largely indirectly on hummingbirds' resources. On the one hand, the finding that species living at higher elevations experienced faster speciation rates is unexpected given that the low temperature and reduced oxygen availability in these areas slows hummingbirds’ rate of molecular evolution [24]. On the other hand, this finding can be explained by the rapid uplift of some portions of the Andes over the past 5 to 8 million years that opened new ecological opportunities for the hummingbirds to diversify [34,68,69]. However, it is important to remember that we found only limited evidence for this positive speciation-elevation relationship.

### (c) Evolutionary divergence, rather than conservatism, relates to speciation

Our results consistently point to evolutionary divergence across multiple niche traits, rather than their conservatism, as a potential driver of hummingbird speciation (figure 2*d–f*). Similar results have been reported for tetrapods [25,29,48,70–72]. However, few previous studies tested for the effects of morphological and niche trait evolution simultaneously, so that their effects have been hard to compare. Morphological traits are often used to test for evolutionary divergence seeking to detect the signal of adaptive radiation [5,48,55], while niche traits are usually analysed to test the conservatism hypothesis [7]. Simultaneously testing both types of traits revealed an effect of niche evolution on hummingbird speciation that is far more consistent than that of morphological evolution (figure 2*d–f*), similar to findings among Furnariidae [30].

Faster evolution of climatic and elevational niche traits among hummingbirds is consistent with the results previously reported for birds as a whole [29,60]. Rapid shifts in niche traits are probably linked to the successful colonization by hummingbirds of the many regions in the Americas over their evolutionary history [34,73]. The group experienced new ecological opportunities as North and Central American species began dispersing between American continents and some lineages colonized the Antilles and the Andes, whose orogenic changes are likely to continue to provide ecological opportunities for speciation [34,69]. For example, the largest shifts in evolutionary rates of temperature position and mid-elevation occurred in the branch leading to two Central American mountain gem sister species (*Lampornis calolaemus* and *L. castaneoventris*) suggesting that movement out of South America contributed to diversification in this lineage (figure 1).

The absence of, or weak evidence for, an association between speciation rates and rates of morphological evolution has been reported by large-scale comparative studies of birds [74,75]. However, those findings contrast with recent work showing that the evolution of other morphological traits, namely plumage [55] and body size [48], influenced hummingbird diversification. Such varying results suggest the relationship between morphological evolution and hummingbird speciation rates depends on the underlying mechanism [75]. For example, plumage colour evolution of males and females hummingbirds are known to relate differently to speciation rates likely as a result of the different roles that colour signalling plays on sexual selection and crypsis [55].

## 5. Caveats and limitations

We acknowledge that our results may be influenced by several factors, including the choice of traits and multiple sources of error, such as Type II errors (i.e. from STRAPP [51]), extinction effects [36,38], and errors associated with estimating species distributions, reconstructing phylogenies and trait measurements. While we cannot exclude these potential errors completely, we accounted for some of the resultant uncertainty by using multiple methods to estimate speciation rates (BAMM, ClaDS, DR) and correlating these estimates with traits and their evolutionary rates using methods that, even though they differ principally in their assumptions and limitations, converged on similar conclusions (Cor-STRATES, PGLS, GLMM, STRAPP, ES-sim) (figure 2). We avoided interpreting the estimates of historical speciation and extinction rates, as these are notoriously hard to reliably tease apart [76] and, instead, focus on present-day estimates of speciation rates near the tips of the hummingbird phylogeny. We used the two most complete phylogenies of hummingbirds published to date [34,38] and the most inclusive geographical distributions [42] after confirming the similarity with an alternative data based on partially different methodology [43]. Since univariate tests (i.e. testing trait effects separately) cannot address trait collinearity, we fitted two classes of multi-predictor regressions, namely PGLS and GLMM (figure 2*b*,*e*; electronic supplementary material, figure S9). Given the wide variety of tests that we conducted, there is some variation across our results, but most findings are qualitatively consistent.

## 6. Conclusion and future directions

Biologists have been fascinated by the dramatic variation in diversity among lineages. In the case of hummingbirds, the bee clade (approx. 36 species) has nine times more species than the topaz clade despite being five times younger (only 5 Myr) [34]. We find that variation in hummingbird diversity is associated with rates of niche evolution and the differences in body mass. These results are consistent with previous work that found the rate of niche evolution to be a better predictor of speciation rates than the rate of morphological evolution [30]. However, the opposite pattern occurs when it comes to the traits themselves, meaning that morphology itself is more likely to play a role in hummingbird speciation than niche-related traits. The difference between the importance of the trait itself and rate of trait evolution is expected based on the key hypotheses in the field. Body mass itself is linked to several mechanisms related to metabolic rates, thermoregulation, competition and foraging that could influence rates of net diversification [10,16], whereas niche evolution corresponds to the classic model of ecospace filling, whereby new diversity is generated as clades expand in the ecospace [1]. Our results support the effects of traits, their rates and niche divergence, and highlight the complexity of mechanisms through which new species are generated, which seem contingent on the type of the trait considered [2]. Thus, our results stress the importance of studying multiple types of traits (morphology, climate, topography) and their evolutionary rates simultaneously [2,25].

## Data Availability

Data and the R script used in this study are available in the electronic supplementary material. The data are provided in electronic supplementary material [77].
